# A multi-country comparative study of two treponemal tests for the serodiagnosis of syphilis amongst men who have sex with men (MSM): Chemo-luminescent assay vs *Treponema pallidum* particle agglutination assay

**DOI:** 10.1186/s12879-024-09100-x

**Published:** 2024-03-14

**Authors:** Lorenzo Gios, Massimo Mirandola, Maddalena Cordioli, Antonella Zorzi, Nigel Sherriff, Jaime Vera, Dominika Wlazly, Mohammed Osman Hassan-Ibrahim, Valeska Padovese, Anabel Darmanin, Rosanna W. Peeling, Magnus Unemo, Karel Blondeel, Igor Toskin

**Affiliations:** 1 Infectious Division of Infectious Diseases, Department of Medicine, Verona University Hospital, Verona, Italy; 2 WHO Collaborating Centre for Sexual Health and Vulnerable Populations - Epidemiology Unit - Division of Infectious Diseases, Department of Medicine, Verona University Hospital, Verona, Italy; 3 School of Sport and Health Sciences, University of Brighton, Brighton, UK; 4 Virology and Microbiology Unit, Department of Pathology and Diagnostics, Verona University Hospital, Verona, Italy; 5 Brighton & Sussex Medical School, University of Sussex and University of Brighton, Brighton, UK; 6 Department of Microbiology & Infection, Royal Sussex County Hospital, University Hospitals Sussex NHS Foundation Trust (Brighton & Haywards Heath Sites), Brighton, UK; 7 Genitourinary Clinic, Department of Dermatology and Venereology, Mater Dei Hospital, Msida, Malta, L-Imsida, Malta; 8 Clinical Research Department, London School of Hygiene & Tropical Medicine, London, UK; 9 Medical Microbiology Department, University of Manitoba, Winnipeg, MB Canada; 10 WHO Collaborating Centre for Gonorrhoea and Other STIs, Department of Laboratory Medicine, Örebro University, Örebro, Sweden; 11 Institute for Global Health, University College London (UCL), London, UK; 12 Department of Sexual and Reproductive Health and Research (includes the UNDP/UNFPA/UNICEF/WHO/World Bank Special Programme of Research, Development and Research Training in Human Reproduction [HRP]), World Health Organization, Geneva, Switzerland; 13 Faculty of Medicine and Health Sciences, Ghent University, Ghent, Belgium; 14 Infectious Diseases Section, Department of Diagnostics and Public Health, University of Verona, Piazzale L. Scuro, 10, 37134 Verona, Italy

**Keywords:** Syphilis, Chemo-luminescent assay, *Treponema pallidum* particle agglutination, MSM

## Abstract

**Introduction:**

International guidelines recommend routine screening for syphilis (aetiological agent: *Treponema pallidum subspecies pallidum*) amongst key populations and vulnerable populations using tests detecting treponemal and non-treponemal antibodies. Whilst treponemal tests have high sensitivities and specificities, they differ regarding subjective or objective interpretation, throughput and workload. Chemiluminescence immunoassays (CLIAs) are cost- and time-effective automated methods for detecting treponemal antibodies. The *Treponema pallidum* particle agglutination assay (TPPA) has been considered the “gold standard” treponemal assay, however, this includes a highly manual procedure, low throughput and subjective interpretation. The present multi-country study evaluated the ADVIA Centaur® Syphilis CLIA (Siemens Healthcare) assay compared to the reference SERODIA-TP·PA® (Fujirebio Diagnostics) for the serodiagnosis of syphilis amongst men who have sex with men (MSM).

**Method:**

1,485 MSM were enrolled in Brighton (UK), Malta, and Verona (Italy) as part of a larger WHO multi-country and multi-site ProSPeRo study. Ethical approval was obtained. Serum was tested with the ADVIA Centaur® Syphilis CLIA assay and SERODIA-TP·PA®, in accordance with the manufacturers’ instructions, for a first round of validation. A second round of validation was carried out for discrepant results that were additionally tested with both Western Blot (Westernblot EUROIMMUN®) and an Immunoblot (INNO-LIA, Fujirebio Diagnostics).

Sensitivity, specificity, positive and negative predictive value (PPV and NPV), likelihood ratios (positive/negative), and the Diagnostic Odds Ratio (DOR)/pre-post-test probability (Fagan's nomogram) were calculated.

**Results:**

Out of 1,485 eligible samples analysed in the first phase, the SERODIA-TP·PA® identified 360 positive and 1,125 negative cases. The ADVIA Centaur® Syphilis CLIA assay (Siemens) identified 366 positives, missclassifying one TPPA-positive sample. In the second phase, the ADVIA Centaur® Syphilis CLIA resulted in 1 false negative and 4 false positive results. Considering the syphilis study prevalence of 24% (95% CI: 22–26.7), The sensitivity of the ADVIA Centaur® Syphilis CLIA assay was 99.7% (95% CI: 98.5–100), and the specificity was 99.4% (95% CI: 98.7–99.7). The ROC area values were 0.996 (95% CI: 0.992–0.999), and both the PPV and NPV values were above 98% (PPV 98.1%, 95% CI: 96.1–99.2; NPV 99.9%, 95% CI: 99.5–100).

**Conclusions:**

The ADVIA Centaur® Syphilis CLIA assay showed similar performance compared to the SERODIA-TP·PA®. Considering the study is based on QUADAS principles and with a homogeneous population, results are also likely to be generalisable to MSM population but potentially not applicable to lower prevalence populations routinely screened for syphilis. The automated CLIA treponemal assay confirmed to be accurate and appropriate for routine initial syphilis screening, i.e. when the reverse testing algorithm is applied.

## Introduction

Syphilis is a sexually transmitted infection caused by *Treponema pallidum subspecies pallidum*. Syphilis remains a major public health concern and during recent decade the incidence has increased, especially in many well-resourced settings and particularly among men who have sex with men (MSM) [1, 2].

International guidelines recommend screening for syphilis, particularly among key populations, including vulnerable sub-groups such as pregnant women, MSM, sex workers, heterosexuals with multiple partners, blood and plasma donors and so on. Given the increasing syphilis incidence, the association with HIV infection, and that the screening algorithms are different across countries worldwide, there is an urgent need to adopt standardised and automated testing procedures to ensure an increased efficiency in testing results delivery.

Traditionally, syphilis diagnostic tests consist of two types of serological assays, namely treponemal and non-treponemal tests. Treponemal tests detect specific treponemal antibodies, whereas non-treponemal tests detect antibodies to cardiolipin, cholesterol and lecithin, which are components of both the treponemal membrane and the membrane of eukaryotic cells. At the same time, it should be underlined that non-treponemal antigen implies a complex issue when testing samples. In fact, cardiolipin is the antigenic lipid in the antigen, cholesterol and lecithin are added after the Pangborn purification studies to improve stability and visibility to the antigenic determinant (therefore resulting in an improved sensitivity of the tests), whilst cardiolipin appears to be more abundant in mitochondria [3].

Treponemal tests include the *Treponema pallidum* hemagglutination assay (TPHA), *Treponema pallidum* particle agglutination assay (TPPA), fluorescent treponemal antibody-absorbed test (FTA-ABS), and different types of more automated enzyme immunoassays or chemiluminescence immunoassays (CLIA). Two common non-treponemal tests regularly used are the Venereal Disease Research Laboratory (VDRL) test and the rapid plasma reagin (RPR) test [3].

Syphilis serological screening algorithms vary across countries. The traditional screening algorithm uses a non-treponemal assay followed by a treponemal test. However, the so-called reversed screening algorithm has become increasingly popular and is currently recommended by many international guidelines [1, 4–6]. In the reversed screening algorithm, a treponemal test is performed first and, if reactive, a different treponemal test and/or a non-treponemal test is carried out to confirm the screening positive result. The non-treponemal test is required to identify active syphilis infections and monitor treatment outcome [7]. Moreover, automated treponemal assays, such as the CLIAs, provide many advantages compared to the traditional manual assays. These methods are less time-consuming and labour-intensive, have higher throughput, and results are objective (no human visual read-out) and with higher sensitivity than non-treponemal tests in the primary and latent disease stages [8, 9]. Due to these advantages, CLIAs are increasingly used as the first treponemal screening test, and manual treponemal tests, such as TPPA, are performed along with non-treponemal test, only when the CLIA is reactive. Over the last few years some studies have been carried out supporting the performance of the treponemal essays (e.g. ADVIA, Bioplex 2200, et al.) for the routine evaluation of samples, including those with potential interfering factors [5]. However, an independent and more extensive evaluation of CLIA performance among a homogeneous population at high risk for this infection, like MSM, and with large sample sizes has not been reported with very recent exceptions [5]. It should be considered in fact that the present study has been designed in the framework of the wider the ProSPeRo project (Project on Sexually Transmitted Infection Point-of-care Testing), a multi-country project supported by the Sexual and Reproductive Health and Research Department of WHO [10]. Therefore, the study presented in this paper has been designed and validated before the publication of the most recent literature in this field [5], when an evaluation study of the performances of CLIA compared to TPPA was needed in order to safely using the automated system instead of a labour-intensive method, as TPPA. According to this the ProSPeRo study, initially designed in 2016, included only manual treponemal tests as reference for the treponemal component of the Point-Of-Care Tests under evaluation. At the same time, a multi-country project like the ProSPeRo study, implying the collection and testing for Syphilis of a large set of specimens for selected populations like MSM, offered the valuable opportunity to plan a validation ancillary study using Chemiluminescence immunoassays (CLIAs) to be compared with the TPPA. In fact, this testing procedure was available as standard process in many of the countries involved in the ProSPeRo consortium and data on its performance was still limited. Another aspect to be considered regarding the importance of having alternative testing methods is the progressive reduced availability in the market of TPPA due to manufacturers’ policy that will make it not available at the end of 2023.

This study compares the performance of the ADVIA Centaur® Syphilis CLIA assay (Siemens Healthcare) compared to the reference TPPA (SERODIA-TP·PA® Fujirebio Diagnostics) and it represents a specific research component of a broader initiative, namely, the ProSPeRo project (Project on Sexually Transmitted Infection Point-of-care Testing), a multi-country project supported by the Sexual and Reproductive Health and Research Department of WHO [10].

## Methods

### Study settings and populations

The objective of this study was to carry out a multi-country concurrent accuracy evaluation of the ADVIA Centaur® Syphilis CLIA assay (Siemens Healthcare) compared to the reference TPPA (SERODIA-TP·PA®; Fujirebio Diagnostics) for syphilis screening (serum samples) amongst a sample of MSM attending the Infectious Disease Department in Verona (Italy), the GenitoUrinary (GU)-clinic in the Mater Dei Hospital, L-Imsida (Malta) and the Brighton & Hove Sexual Health and Contraception Service (SHAC) in Brighton (UK). Using the same protocol and criteria, an additional sample of patients (MSM) was enrolled at the Verona site in the framework of an EU-funded project called Sialon [11].

#### Inclusion criteria

Criteria were adopted in line with the ones used in the context of the ProSPeRo study [10], namely: i) being a man who has sex with men who asks for an HIV and syphilis test or accepts to being tested for HIV and syphilis, as proposed by the interviewer, on the basis of the information provided during the routine counselling interview; ii) being older or equal to 18 years old, and iii) providing a written informed consent to take part in the study.

#### Exclusion criteria

MSM who had previously participated in the study were considered as not eligible, as well as MSM who did not provide written consent or were younger than 18 years.

### Study procedure

#### Recruitment, enrolment, and consent

For each site, MSM were recruited consecutively, when presenting to the clinic. If the potential participant complied with the inclusion and exclusion criteria, written informed consent was collected.

#### Specimen collection

For each participant, a blood sample was obtained. The blood sample (10 ml in tube) was collected by venepuncture in line with the local standard procedures. All specimens were stored in tubes and labelled with a unique bare code number, to allow for linking different data of the same participant.

After performance of the ProSPeRo reference tests [10], each reference laboratories in Brighton and Malta froze remaining sera from each participant at -80 °C until being shipped to the Microbiology Unit of the University of Verona, which was selected as the unique reference laboratory for this exercise and where the CLIA Siemens ADVIA Centaur® Syphilis assay was performed.

#### Testing materials

Trained staff processed and tested the specimens and interpreted the results according to the manufacturers’ instructions and recorded the results on a specific form.

All sera samples were tested qualitatively with the CLIA Siemens ADVIA Centaur® Syphilis assay and quantitatively with the TPPA SERODIA-TP·PA®, following the manufacturers’ instructions. Discordant samples were subsequently tested with both Western Blot (Westernblot EUROIMMUN®) and an Immunoblot (INNO-LIA, Fujirebio Diagnostics).

According to the original protocol, all samples were also tested with a non-treponemal test (RPR, Bio-Rad Laboratories).

**Fig. 1:**
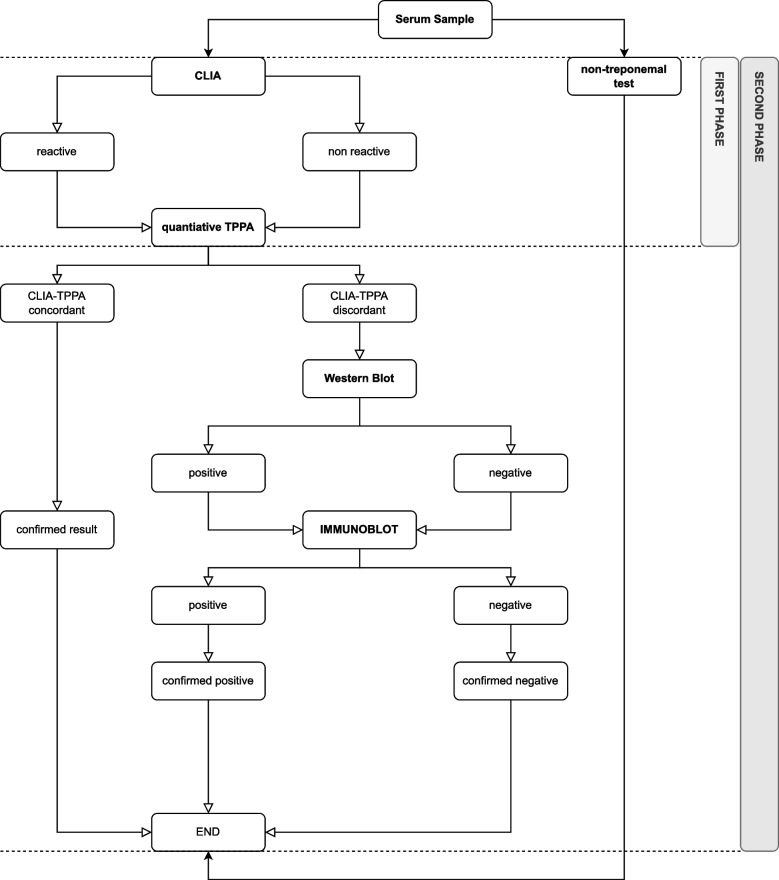
Testing algorithm and the two phases of the validation study

Samples were tested according to the algorithm presented in Fig. 1.

#### Index test

The result of the index test was based on the CLIA Siemens ADVIA Centaur® Syphilis assay.

#### Reference test

In the context of this validation study, two phases were designed:

i) the performance of the ADVIA Centaur® Syphilis CLIA assay was assessed considering only the results from the SERODIA-TP·PA®;
ii) the performance of the ADVIA Centaur® Syphilis CLIA assay was evaluated considering the results from the SERODIA-TP·PA® and an additional testing for the discordant cases using both Western Blot (Westernblot EUROIMMUN®) and an Immunoblot (INNO-LIA, Fujirebio Diagnostics) testing.

Therefore, the reference test for the first phase was based only on TPPA SERODIATP·PA®, that is, considering only the first part of the algorithm (see Fig. 1).

In the second phase, the reference test (outcome variable) was considered positive or negative according to the final result of the entire algorithm (see Fig. 1).

#### Follow-up of participants

Pre- and post-test counselling was provided to all participants according to WHO recommendations and local clinical practice. Patients with confirmed positive results were treated according to the standards of care described in national guidelines for each evaluation site.

### Sample size

The sample size was determined according to the core protocol, the WHO promoted, HIV/syphilis point-of-care test (POCT) evaluation study (independent clinic-based evaluation of dual POCTs for screening of HIV and syphilis in MSM in Italy, Malta, Peru and the UK). Under these sample sizes ranges, specific calculations were performed to explore the width of 95% confidence interval for the estimation of sensitivities of the CLIA in comparison with TPPA. The formula used for the sample size calculation was based on the 2006 WHO/TDR expert panel document on the evaluation of new diagnostic methods and techniques [12].

### Data analysis

Data analysis was guided by QUADAS-2 principles [13], whilst statistical analyses were performed with STATA 17 (StataCorp, College Station, Texas, United States). The DIAGT routine developed by Seed [14] was used to estimate sensitivity, specificity, predictive positive and negative values, positive and negative likelihood ratios, and Diagnostic Odds Ratio (DOR).

Sensitivity was defined as the proportion of positive test results among the individuals with treponemal antibodies (reference Test). Specificity was defined as the proportion of negative test results among individuals without a treponemal antibodies (reference Test).

The Positive Likelihood Ratio (PLR) measures how frequently a positive test is found in infected vs. non-infected individuals. On the other hand, the Negative Likelihood Ratio (NLR) measures how likely a negative result is in infected vs. non-infected individuals.

The odds of a positive result in infected individuals compared to the odds of a positive result in non-infected individuals is calculated according to the formula: (True Positive / False Negative) / (False Positive/True Negative). DOR depends significantly on the sensitivity and specificity of a test. The area under the curve (AUC) is a global measure of test performance, with a value of 1 indicating complete accuracy.

Although the testing was carried out in one lab (Verona University Hospital – Virology and Microbiology department), in order to assess pooled sensitivity and specificity with relative 95% confidence intervals, a random-effects model was used to pool the estimated effects using the METADTA routine developed by Nyaga & Arbyn [15]. The random effects model was used to estimated heterogeneity of the true effect sizes. Summary Receiver-Operating Characteristics (SROC) and Forestplot graphs were used to describe summary points and their confidence and prediction regions. Between-study heterogeneity was evaluatd using the I2 statistics by Zhou & Dendukuri [16].

Finally, a Diagnostic Odds Ratio (DOR; pre-post-test probability) was calculated using Fagan's nomogram to provide evidence for clinicians when determining the probability of a patient truly having a condition of positivity considering the results of the examined test.

## Results

A detailed description of the study population is presented in the paper reporting the results from the independent clinic-based evaluation of dual POCTs for screening of HIV and syphilis in MSM in Italy, Malta, Peru and the United Kingdom [10].

As mentioned, in the first phase of the validation, the performance of the ADVIA Centaur® Syphilis CLIA assay (Siemens) was assessed considering only the results from the SERODIA-TP·PA®. In the second validation phase, the performance of the ADVIA Centaur® Syphilis CLIA assay was evaluated considering the entire testing algorithm (SERODIA-TP·PA® results plus testing of the discordant cases with both Western Blot and Immunoblot).

### First phase – Performance of the ADVIA Centaur® Syphilis CLIA assay compared to the reference SERODIA-TP·PA®

Out of 1,485 eligible samples analysed in the first phase, the SERODIA-TP·PA® identified 360 (24.24%) positive and 1,125 negative cases. The ADVIA Centaur® Syphilis CLIA assay identified 366 (24.64%) positives, 7 of which were misclassified (1.91%). When considering the cases reported as negative with the TPPA testing, 1.118 were correctly identified as negative using the CLIA, whilst 1 was misclassified (0.09%). (Table 1).

**Table 1:** SERODIA-TP·PA® (reference test) versus ADVIA Centaur® Syphilis CLIA assay (index test) results

SERODIA-TP·PA®	ADVIA Centaur® Syphilis CLIA assay		
(Reference test^a^)	(Index test)		
	**Positive**	**Negative**	**Total**
**Positive**	359	1	360
**Negative**	7	1,118	1,125
**Total**	366	1,119	1,485

^a^only phase 1 of the testing algorithm was considered (Fig. 1)

For this comparison, only the first part of the testing algorithm was considered (see Fig. 1).

The prevalence of treponemal antibodies based on TPPA testing was 24.24% (95% CI: 22.12%—26.48%). The sensitivity, specificity, PPV, NPV, and ROC area are summarised in Table 2.

**Table 2:** Reference test performance in terms of sensitivity, specificity, ROC area and PPV-NPV (Study prevalence of treponemal antibodies: 24%)

	**value**	**95% CI**	
Prevalence	24.2	22.1	26.5
Sensitivity	99.7	98.5	100
Specificity	99.4	98.7	99.7
ROC area	0.996	0.992	0.999
PPV	98.1	96.1	99.2
NPV	99.9	99.5	100

The sensitivity of the ADVIA Centaur® Syphilis CLIA assay was 99.7% (95% CI: 98.5–100), and the specificity was 99.4% (95% CI: 98.7–99.7). The ROC area values were 0.996 (95% CI: 0.992–0.999), and both the PPV and NPV values were above 98% (PPV 98.1%, 95% CI: 96.1–99.2; NPV 99.9%, 95% CI: 99.5–100).

### Second phase – Performance of the ADVIA Centaur® Syphilis CLIA assay compared to the reference SERODIA-TP·PA® and Western Blot / Immunoblot for discordant cases

In the second phase, the performance of the ADVIA Centaur® Syphilis CLIA assay was evaluated using the results of the entire testing algorithm presented in Fig. 1. This means that the reference test (final outcome variable) was considered positive or negative according to the final result of the entire algorithm, including the testing of the discordant cases with both Western Blot (Westernblot, EUROIMMUN®) and an Immunoblot (INNO-LIA, Fujirebio Diagnostics). Results are presented in Table 3.

**Table 3:** SERODIA-TP·PA® versus ADVIA Centaur® Syphilis CLIA assay results, including the testing of discordant cases with the complete testing algorithm

**SERODIA-TP·PA® + complete testing algorithm**	ADVIA Centaur® Syphilis CLIA		
(Reference test^a^)	(Index test)		
	**Positive**	**Negative**	**Total**
**Positive**	362	1	363
**Negative**	4	1,118	1,122
**Total**	366	1,119	1,485

^a^Complete testing algorithm phase 1 and phase 2, Fig. 1: (SERODIA-TP-PA + Western Blot + Immunoblot)

Out of 1,485 eligible samples analysed in the second phase, the complete algorithm (SERODIA-TP·PA® + Western Blot + Immunoblot) identified 363 positive (24.44%) and 1,122 negative cases (75.56%). As reported above, ADVIA Centaur® Syphilis CLIA assay (Siemens) identified in total 366 (24.64%) positive samples, however 4 of them were misclassified as positive (but negative to the Reference Test). One sample was classified as negative (0.09%) by the index test while it resulted positive to the complete algorithm Reference Test. 1,118 (99.91%) were correctly identified classified as negative.

When considering the results of the second phase of the validation study (Table 4), the sensitivity of the ADVIA Centaur® Syphilis CLIA was 99.7% (95% CI: 98.5–100), and the specificity was 99.6% (95% CI: 99.1–99.9). The ROC area values were 0.997 (95% CI: 0.994–1), and both the PPV and NPV values were above 98% (PPV 98.9%, 95% CI: 97.2–99.7; NPV 99.9%, 95% CI: 99.5–100).

**Table 4:** Reference test performance in terms of sensitivity, specificity, ROC area and PPV-NPV (Study prevalence of treponemal antibodies: 24%)

	**value**	**95% CI**	
Prevalence	**24**	22	26.7
Sensitivity	99.7	97.9	100
Specificity	99.5	98.8	99.8
ROC area	0.997	0.994	1
PPV	98.9	97.2	99.7
NPV	99.9	99.5	100

**Fig. 2:**
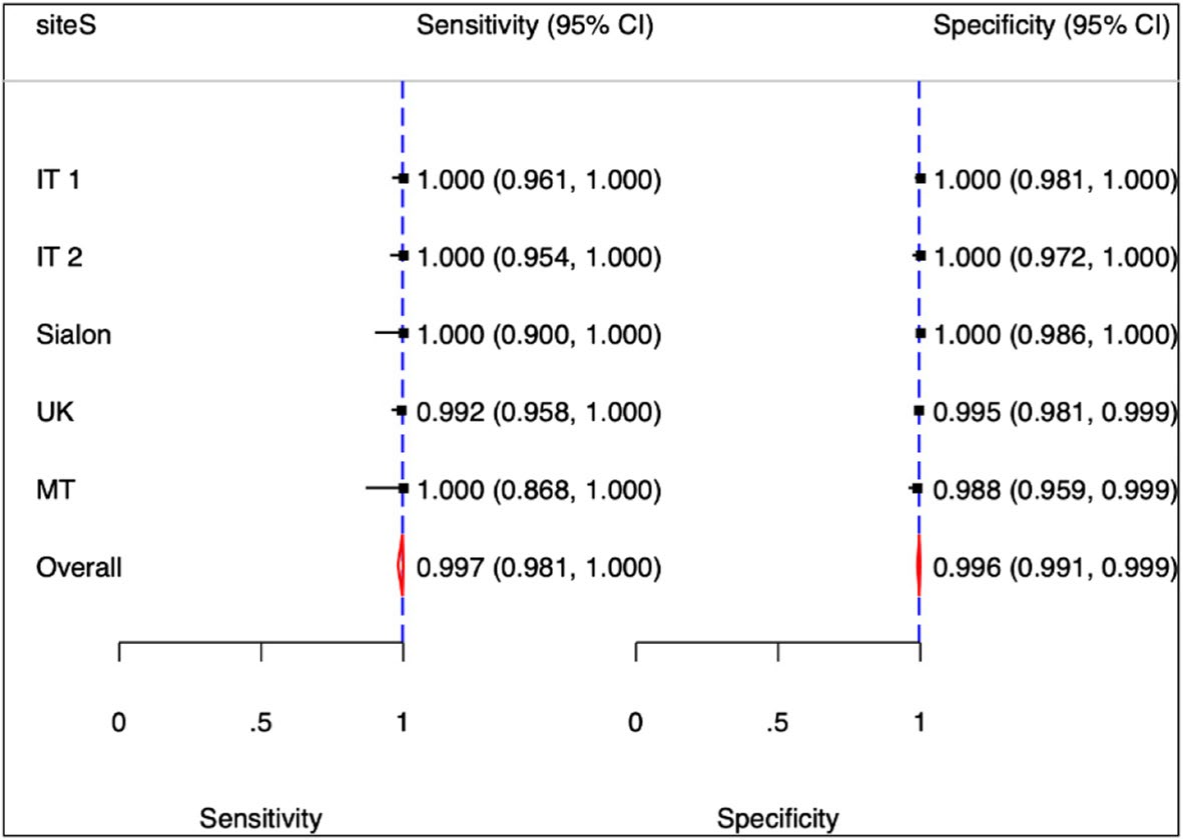
Study specific test accuracy (absolute measures)

**Fig. 3:**
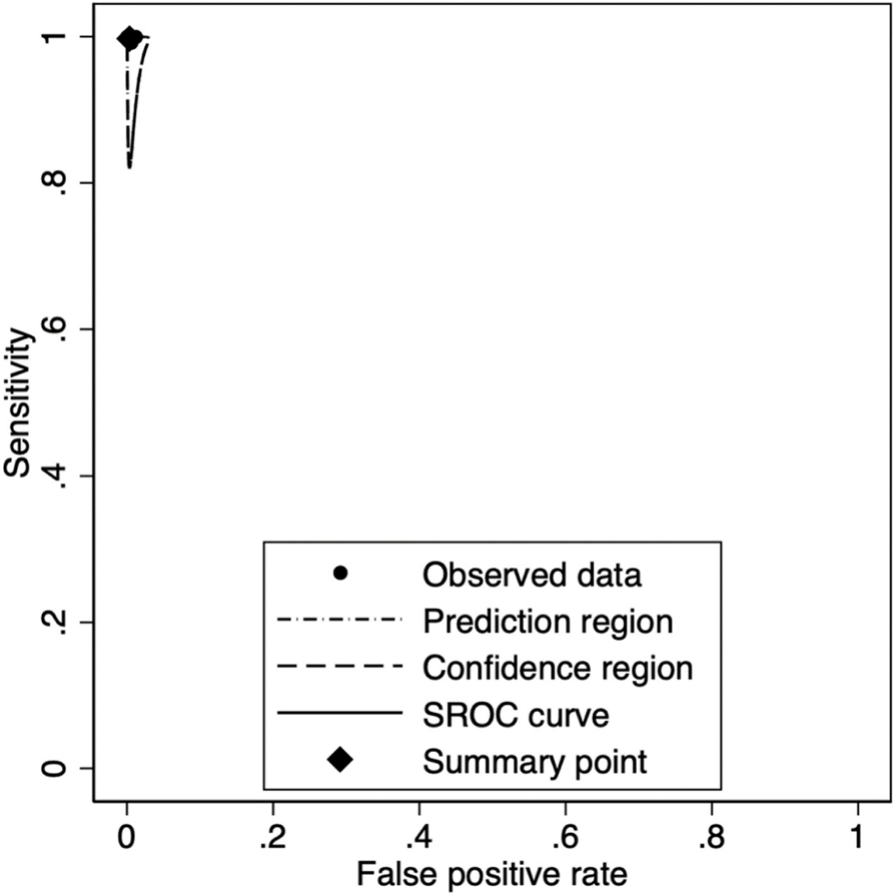
Summary Receiver-Operating Characteristics (SROC) with confidence and prediction regions

Whilst inter-lab variation was to be excluded due to the centralised laboratory for samples testing, participants of each site were considered a potential source of variation that could not be excluded a priori and sites were considered a proxy of individual variation. A meta-analytic approach was adopted considering the sites as random component (Table 5). The Likelihood Ration (LR) test comparing Random Effect (RE) versus Fixed Effect (FE) models issued a not significant result (Chi2 = 0.000. 2 df *p* = 1.0000) and therefore this potential source of variation was excluded. Figures 2 and 3 present the estimates of the sensitivity and specificity for each study site with the overall effect and Summary Receiver-Operating Characteristics (SROC) with confidence and prediction regions.

**Table 5:** Study specific test accuracy (absolute measures)

Study	Estimate	95% Conf. Interval		Estimate	95% Conf. Interval	
IT 1	1.000	0.961	1.000	1.000	0.981	1.000
IT 2	1.000	0.954	1.000	1.000	0.972	1.000
Sialon	1.000	0.900	1.000	1.000	0.986	1.000
UK	0.992	0.958	1.000	0.995	0.981	0.999
MT	1.000	0.868	1.000	0.988	0.959	0.999
Overall	0.998	0.971	1.000	0.997	0.985	0.999

LR Test: RE vs FE model Chi2 = 0.000 with 2 degrees of freedom and a *p*-val = 1.0000

### Discordant cases

An overview on discordant cases emerged from the first phase of the evaluation (8 cases) is represented in Table 6, with a detailed presentation in Table 7 including signs, symptoms, and previous infections history. Discordance was analysed considering the three levels of testing adopted for the validation exercise, namely i) the ADVIA Centaur® Syphilis CLIA assay, ii) the SERODIA-TP·PA® and ii) the additional testing with both Western Blot (Westernblot EUROIMMUN®) and an Immunoblot (INNO-LIA, Fujirebio Diagnostics). The non-treponemal test (RPR) adopted as standard component of the study was also considered. It should be underlined that information related to signs, symptoms, and previous infections history was examined only for the discordant cases, in order to enrich the amount of details for these specific situations. Although they were further investigated to provide the reader with more information on the clinical component, the reference test is only based on the WB/Immunoblot testing results.

**Table 6:** Overview of discordant results and related testing

N	ADVIA Centaur® Syphilis CLIA assay	Complete algorithm	Titre	Non Trep testing	WB	Immunoblot
1	Positive	Negative	0	0	Negative	Negative
2	Positive	Negative	0	0	Positive	Positive
3	Negative	Positive	160	0	Positive	Positive
4	Positive	Negative	0	0	Negative	Negative
5	Positive	Negative	0	0	Negative	Negative
6	Positive	Negative	0	0	Positive	Positive
7	Positive	Negative	0	0	Negative	Negative
8	Positive	Negative	0	0	Positive	Positive

**Table 7:** Details overview of the discordant results

ProSPeRo										Before enrollment					Interpretation
N	Enrollment date	Syphilis signs/symptoms	Local treponemal screening test: type, date, result	Local treponemal confirmatory test: type, date, result	Verona CLIA result	Verona TPPA result	Verona RPR result	Verona WB (interpretation)	Verona IB (interpretation)	HIV serostatus	Previous syphilis diagnosis (year)	Treponemal tests at diagnosis: type, date, result (carried out at local level)	Syphilis test: type, date, result (carried out at local level)	Syphilis signs/symptoms at first screening	CLIA result
1	28/11/19	No	TPHA, 11/2019, negative	NA	pos	neg	neg	TpN17 ± (neg)	TpN17 + , TpN15 ± TmpA ± (pos)	pos (2002)	Yes (2017)	NA	EIA 06/20, pos, VDRL 1:16, TPHA 1:10240	Yes	True positive
2	25/09/18	No	CLIA, 01/18, pos	TPHA, 01/18, 1:80	pos	neg	neg	TpN47 + (indet)	TpN47 + , TpN17 + (pos)	neg	Yes (2017)	TPHA, 17, 1:640	EIA, 04/19, pos, VDRL 1:256	Yes	True positive
3	02/10/18	No	CLIA, 10/18, neg	NA	neg	pos	neg	neg	Neg	neg	No	NA	EIA, 10/19, neg	NA	True negative
4	13/08/18	No	CLIA, 08/18, neg	NA	pos	neg	neg	neg	Not performed (no leftover)	neg	No	NA	EIA, 04/19, neg	No	False positive
5	01/03/19	No	CLIA, 03/19, neg	NA	pos	neg	neg	neg	TpN17 ± (neg)	pos	No	NA	EIA, 12/20, neg	No	False positive
6	27/03/19	No	CLIA, 03/19, neg	NA	pos	neg	neg	neg	TpN17 + , TmpA ± (pos)	pos	No	NA	EIA 12/20, neg	No	Probable previous (undiagnosed) infection
7	25/11/19	No	TPHA, 11/2019, negative	NA	pos	neg	neg	TmpA ± (neg)	TpN17 + TmpA + (pos)	pos (05/2015)	Yes (2015)	TPHA, 15, 1:160	EIA 07/20, neg	No	True positive
8	06/09/18	No	NA	TPHA, 09/18, 1:80	pos	neg	neg	neg	TpN47 + TpN17 + + (pos)	neg	Yes (2004)	TPHA, 04, 1:80	NA	NA	True positive

In one case the ADVIA Centaur® Syphilis CLIA testing resulted negative, whilst the entire algorithm provided a positive result (titre 160). The case was reported as positive considering the WB/Immunoblot testing. The difference between entire algorithm including TPPA and chemiluminescence tests might be related to the fact that TPPA targets all the surface antigens, whilst chemiluminescence testing targets a limited number of antigens. When considering samples resulted as false positive, considering Immunoblot results and clinical information on the specific patients, these discordant cases were defined as *past infections*.

Finally, it cannot be excluded that an impact on discordant cases might be somehow linked with the difference between the antigenic composition of the two treponemal tests assessed in the present study.

### Diagnostic Odds Ratio (DOR)—pre-post test probability

**Fig. 4:**
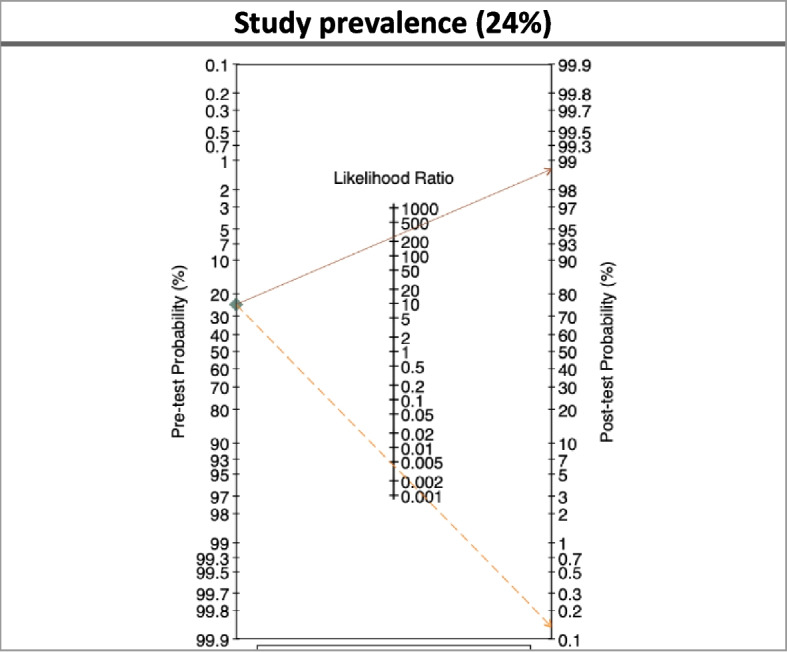
Pre & Post Test Probability Fagan's Bayesian nomogram based the study prevalence of treponemal antibodies (24%). Continuous line LR positive / Dash line LR Negative

Positive and negative likelihood ratio (LR) were calculated using the syphilis prevalence found in the study (24%). LR illustrate the probability that—for a specific patient—treponemal antibodies for active or previous syphilis were or were not detected The Diagnostic Odds Ratio (DOR) for pre-post test probability was graphically shown using the Fagan's nomogram (Fig. 4). As shown, the probability of correctly classifying a positive case is 99%, while the probability of misclassifying a negative case is below 0.1%. However, results need to be interpreted with caution as the Fagan's nomogram has limited accuracy and the pre- and post-test ranges of the nomogram are limited (generally from 0.001 to 0.990).

### Scenarios of prevalence of treponemal antibodies

**Fig. 5:**
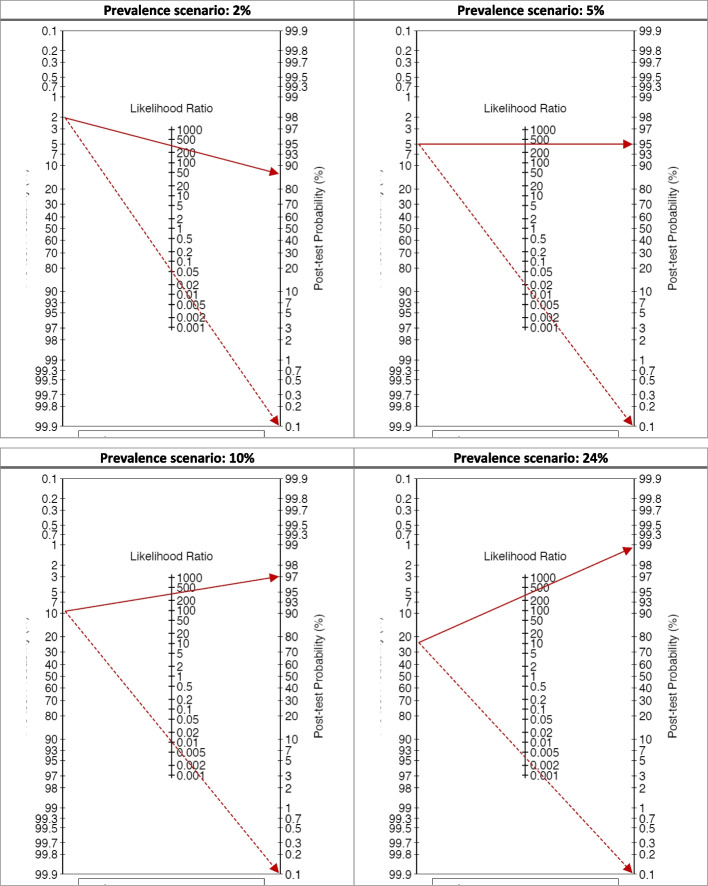
Pre & Post Test Probability Fagan's Bayesian nomogram based on different scenarios of prevalence of treponemal antibodies. Continuous line LR positive / Dash line LR Negative

Considering the potential application of the test in different contexts and settings, its performance was calculated in relation to different prevalence scenarios. In particular, the following scenarios of prevalence of treponemal antibodies were considered: 2%, 5%, 10%, and 24% (see Table 8), considering also the potential prevalence reported in the literature for this type of population [17]. For each scenario, specificity and the sensitivity are calculated. While the specificity and the sensitivity of the test are not influenced by the prevalence, predictive values ​​vary in relation to the prevalence reported in the specific target population. In addition, pre & post Test Probability Fagan's Bayesian nomogram is calculated on the basis of different prevalent scenarios (see Fig. 5).

**Table 8:** Prevalence’s scenarios

	**value**	**95% CI**		**value**	**95% CI**		**value**	**95% CI**		**value**	**95% CI**	
Prevalence	**2%**	-	-	**5%**	-	-	**10%**	-	-	**24%**	-	-
Sensitivity	99.7%	98.5	100	99.7%	98.5	100	99.7%	98.5	100	99.7%	98.5	100
Specificity	99.4%	98.7	99.7	99.4%	98.7	99.7	99.4%	98.7	99.7	99.6%	99.1	99.9
ROC area	0.996	0.992	0.999	0.996	0.992	0.999	0.996	0.992	0.999	0.997	0.994	1
PPV	76.6%	59.9	86.2	89.4%	79.4	94.1	94.7%	89.1	97.1	98.9%	96.9	99.5
NPV	100%	100	100	100%	99.9	100	100%	99.8	100	99.9%	99.4	100

The sensitivity of the ADVIA Centaur® Syphilis CLIA assay was 99.7% (95% CI: 99.7–100), and the specificity was 99.6% (95% CI: 99.1–99.9), and they do not vary as expected when different prevalence scenarios are applied. Area under the curve (ROC) value was 0.997 (95% CI: 0.994–1). When prevalence of treponemal antibodies was 2%, the PPV was 76.6% (95% CI: 59.9–86.2) and NPV was 100% (100% CI: 100–100). When considering the measured prevalence of 5%, the PPV and NPV remained at 89.4% (95% CI: 79.4–94.1) and 100% (95% CI: 99.9–100), respectively. In case of a prevalence of 10%, PPV would be 94.7% (95% CI: 89.1–9701) and NPV 100% (95% CI: 99.8–100), whilst 24% prevalence would result in 98.9% (95% CI: 96.9–99.5) and 99.9% (95% CI: 99.4–100), respectively.

The Diagnostic Odds Ratio (DOR) for pre-post-test probability was used also for the different prevalence scenarios, adopting the Fagan's nomograms (see Fig. 5).

### Clinical implications

In terms of clinical implications, the ADVIA Centaur® Syphilis CLIA assay may be slightly more sensitive compared to SERODIA-TP·PA®, whilst for the negative cases both testing techniques were reliable. In one case, the patient was confirmed as a true negative case and one case appeared to be a probable previously undiagnosed decapitated infection. Considering specificity, this could also be considered with caution as false positive Syphilis diagnosis could lead to potentially critical implication, particularly in cases of pregnant women as target population for this type of testing.

CLIA sensitivity seems to be equivalent to the TPPA test. Therefore, from a clinical point of view, the two tests seem to provide the same valuable information. In a critical case, the positivity reported through ADVIA Centaur® Syphilis CLIA assay testing is related with a previous infection that was arguably treated adequately. Presumably, the adequacy of the treatment has led – over the time – to the lack of antibodies detected by the SERODIA-TP·PA®.

## Discussion

The present multi-country study is part of the broader ProSPeRo initiative [10] and it has been designed to specifically compare the performance of the ADVIA Centaur® Syphilis CLIA assay versus the reference SERODIA-TP·PA® for syphilis screening amongst MSM. The objective was to provide evidence supporting the use of the automated CLIA as an alternative to the TPPA for treponemal antibody detection, at least among high-prevalence MSM populations. This could be considered as the first independent multi-centre and multi-country validation study in this field.

Considering that many European countries already use automated CLIA assays or similar high-throughput assays for initial screening, this study confirms this automated method as viable testing approach to routine initial syphilis screening. This approach has many advantages when compared to the more labour-intensive TPPA testing, particularly in high-volume laboratories, in settings where labour costs are high. In addition, this study might be particularly useful for the independent validation considering the use of samples from a homogeneous population such as MSM, with a higher prevalence level compared to the general population. Finally, the present study contributes in widening the knowledge on the automatic treponemal test (CLIA) performance in the field. This could lead to a potential paradigm shift toward automated technologies in testing for screening, that is, replacing TPPA by CLIA, as it is a non-subjective, less time-consuming and labour-intensive method.

One of the limitations of the present validation study is that it was designed before the publication of the most recent literature in this field [5]. In fact, in 2016 an evaluation study specifically targeting the performances of CLIA compared to TPPA was needed in order to justify the use of automated systems rather than implementing more labour-intensive methods such as TPPA. In addition, for the target population considered for this exercise an high prevalence has been reported, leading to very few false positives with the CLIA, a pattern that seems to differ from prior studies in the literature on antenatal populations as well as older studies using TrepSure, Bioplex, and LIAISON, which included mixed populations [18–20]. In particular, the study by Mmeje and colleagues found that women who were isolated CLIA positive and RPR negative, a negativity in TPPA would often serorevert their tests back to CLIA negative upon repeat testing. In case of a scenario where an algorithm of CLIA as a first line testing and an RPR as second line, followed by an additional testing using CLIA, this could lead to a misclassification of disease status leading to overtreatment.

An additional limitation is linked with the specific target population considered for this study, namely MSM, and the related prevalence in this population. Whilst results might be generalizable to MSM, generalization might not be applicable to lower prevalence populations routinely screened for syphilis, such as pregnant women.

Nevertheless, the available knowledge and the valuable opportunity provided by the concomitant and wider ProSPeRo initiative made the outcomes of this study a valid contribution to improve knowledge both in terms of different testing approaches for serodiagnosis and different prevalence scenarios of treponemal antibodies.

An additional limitation is represented by the number of samples considered. Even if the study is the first independent evaluation with clinical samples and sufficient positive cases to evaluate the performance of the ADVIA Centaur® Syphilis CLIA assay compared to the SERODIA-TP·PA®, larger sample sizes could provide more accurate estimates in terms of performance of the test.

However, to reduce biases and limitations related to the methodological asset of the study, this evaluation protocol was designed to minimise inter-lab variation, as the Microbiology Unit of the University of Verona was adopted as the only reference laboratory for this study. In addition, methodological and operational procedures have been designed on the basis of QUADAS principles and samples were collected from a homogeneous population (MSM populations with a higher prevalence level compared to the general population in all sites included). This methodological asset leads to an improved results’ generalisability.

## Conclusion

In conclusion, this multi-country study, conducted as part of the ProSPeRo initiative, aimed to evaluate the performance of the ADVIA Centaur® Syphilis CLIA assay compared to the SERODIA-TP·PA® for syphilis screening among MSM populations. The findings suggest that automated CLIA testing could serve as a viable alternative to TPPA, particularly in high-prevalence MSM populations. The study contributes to expanding knowledge on automated treponemal testing and highlights the potential for a paradigm shift towards automated technologies in screening. Despite the limitations of the initiative, the study brings scientific evidence to consider changes to routine laboratory algorithms for syphilis. At the same time, it should be underlined that i) the testing algorithm is to be used for samples of serum and/or blood and ii) that this approach cannot be considered as a viable option for other purposes and diagnostic scenarios, as for instance for diagnosis of neurosyphilis.

To conclude, findings suggest that the automated ADVIA Centaur® Syphilis CLIA assay is viable and reliable testing approach for routine initial syphilis screening when the reverse testing algorithm is used.

## Data Availability

Availability on request.

## Abbreviations

AIDS: Acquired Immune Deficiency Syndrome
CLIA: Chemo-luminescent Assay
HIV: Human Immunodeficiency Virus
MSM: Men who have Sex with Men
POCT: Point-of-Care Test
ProSPeRo: Project on Sexually Transmitted Infection Point-of-Care Testing
RDT: Rapid Diagnostic Test
RPR: Rapid Plasma Reagin
STIs: Sexually Transmitted Infections
TPPA: Treponema Pallidum Particle Agglutination assay
TPHA: Treponema Pallidum Hemagglutination assay
WHO: World Health Organization
